# Antimicrobial Susceptibility Profiles of *Escherichia coli* Isolates from Clinical Cases of Chickens in Hungary Between 2022 and 2023

**DOI:** 10.3390/antibiotics14020176

**Published:** 2025-02-11

**Authors:** Ákos Jerzsele, Ábel Szabó, Franciska Barnácz, Bence Csirmaz, László Kovács, Ádám Kerek

**Affiliations:** 1 Department of Pharmacology and Toxicology, University of Veterinary Medicine, István Utca 2, H-1078 Budapest, Hungary; jerzsele.akos@univet.hu (Á.J.); szabo.abel@student.univet.hu (Á.S.); barnacz.franciska@student.univet.hu (F.B.); csirmaz.bence@student.univet.hu (B.C.); 2 National Laboratory of Infectious Animal Diseases, Antimicrobial Resistance, Veterinary Public Health and Food Chain Safety, University of Veterinary Medicine, István Utca 2, H-1078 Budapest, Hungary; kovacs.laszlo@univet.hu; 3 Department of Animal Hygiene, Herd Health and Mobile Clinic, University of Veterinary Medicine, István Utca 2, H-1078 Budapest, Hungary; 4 Poultry-Care Kft., Lehel út 21, H-5052 Újszász, Hungary

**Keywords:** *Escherichia coli*, antimicrobial resistance, AMR, minimum inhibitory concentration, MIC, poultry, chickens, clinical strains, Hungary

## Abstract

**Background**: The global spread of antimicrobial resistance (AMR) necessitates collaborative approaches between animals, their environment, and public health sectors, fostering the One Health concept. *Escherichia coli* (*E. coli*) is a zoonotic, facultative pathogenic bacterium. Its public health significance underlines the need for the regular monitoring of different strains causing clinical disease, especially in poultry, more specifically in chickens, which have become a critical source of animal protein. **Methods**: The antimicrobial susceptibility of 133 *E. coli* strains isolated from clinical cases in large-scale Hungarian poultry between 2022 and 2023 was assessed via the gold-standard minimum inhibitory concentration (MIC) determination, which provides internationally comparable results. **Results**: Our findings revealed high resistance levels to widely used antibiotics, including amoxicillin (57.9%), neomycin (78.9%), doxycycline (46.6%), and potentiated sulfonamides (43.6%). Resistance to these critically important antibiotics is particularly concerning due to their public health significance. Comparison with regional human resistance data revealed similar patterns for β-lactam antibiotics; however, aminoglycosides, fluoroquinolones, and potentiated sulfonamides exhibited significantly higher resistance levels in veterinary isolates. **Conclusions**: Our results highlight the importance of routine surveillance, which has both veterinary and public health implications. Future efforts to correlate antibiotic usage with resistance patterns and to elucidate the genetic background of multidrug-resistant strains will further strengthen the One Health approach.

## 1. Introduction

Antimicrobial-resistant bacteria observed in food-producing animals can be transmitted to humans via the food chain, which is of particular importance for *Escherichia coli* (*E. coli*), a prominent zoonotic bacterium. Its dissemination is also facilitated by water, various environmental contaminants, and direct contact with animals. In people, infections caused by resistant bacteria can result in treatment failures, necessitating the use of second-line antibiotics (e.g., fluoroquinolones) during therapy. Furthermore, commensal gut microbiota may act as reservoirs of resistance genes, which can subsequently be transferred to other animals and humans [1]. Avian-derived *E. coli* pathotypes can cause a range of clinical symptoms in humans, including gastroenteritis, urinary tract infections, and, in severe cases, septicemia [2].

In light of this, the European Commission adopted an action plan in 2017 to combat antimicrobial resistance (AMR), emphasizing the One Health approach, which addresses veterinary and human resistance simultaneously. The objectives include promoting the prudent use of antimicrobials, improving infection prevention strategies, and strengthening monitoring and surveillance efforts [3]. This is achieved through continuous data collection, analysis, and reporting, which help establish temporal trends [4]. The European Centre for Disease Prevention and Control (ECDC), the European Medicines Agency (EMA), and the European Food Safety Authority (EFSA) regularly produce reports on AMR [5,6,7]. The monitoring of animals, particularly poultry, focuses on commensal *E. coli* and the assessments mandate the monitoring of extended-spectrum β-lactamase (ESBL), *ampC* gene presence, and carbapenemase production. For these analyses, microdilution methods are prescribed, and the results are interpreted using the European Committee on Antimicrobial Susceptibility Testing (EUCAST) breakpoints and epidemiological cut-off values (ECOFF) [8].

The harmonization of AMR data derived from clinical isolates is critical for veterinary medicine. Certain programs, such as VetPath, ComPath, and MycoPath, operate under the management of the European Animal Health Study Centre (CEESA) in collaboration with pharmaceutical companies to harmonize AMR data from food-producing and companion animals. However, this effort does not yet provide a fully unified European perspective [9]. Across Europe, individual countries have developed their own surveillance systems to monitor AMR, including the Czech Republic [10], Denmark [6], Finland [11], France [12], Germany [13], Ireland [14], Norway [15], Sweden [16], and the United Kingdom [17].

Poultry and poultry products pose a significant risk for the dissemination of *E. coli* strains, particularly in spreading plasmid-mediated colistin resistance at the European level [18]. Research conducted in Nigeria revealed that multidrug-resistant (MDR) *E. coli* isolates are widespread in poultry farming environments and the marketplaces where these products are sold. The emergence of MDR *E. coli* with a new sequence type was observed in two isolates, suggesting plasmid-mediated transmission [19]. In Thailand, extended-spectrum β-lactamase-producing *E. coli* (ESBL) strains were detected in backyard poultry farms in 2020, underscoring the role of small-scale farms as a potential source of MDR *E. coli* strains [20]. Plasmid-mediated resistance mechanisms, such as those conferred by the *mcr-1* gene, have been identified as significant contributors to multidrug resistance in *E. coli*, complicating treatment efforts [18]. The similarity between human extraintestinal pathogenic *E. coli* (ExPEC) strains and avian pathogenic *E. coli* (APEC) strains in their pathogenic roles is supported by research, indicating that these strains share a common bacterial ancestor, reflecting a shared evolutionary origin [21].

According to a Chinese study, 94% of *E. coli* isolates from poultry exhibited resistance to at least one antimicrobial agent, while 83% were resistant to at least three different antimicrobial classes, with the most common resistances being to tetracycline, nalidixic acid, and sulfamethoxazole [22]. Another study in the United States found that *E. coli* isolates recovered from fresh poultry manure used in vegetable farming were completely resistant to tetracycline, erythromycin, cefoxitin, and streptomycin [23]. In Germany, research conducted between 2014 and 2017 revealed that resistance to ampicillin, gentamicin, and tetracycline occurred less frequently in clinical isolates from turkeys compared to those from broiler chickens [24]. This further supports the necessity of conducting species-specific and utilization-based surveillance studies, as differing rearing periods inherently lead to variations in both the types and quantities of antibiotics used. Global One Health initiatives are increasingly driven by research emphasizing the role of wild birds in maintaining and disseminating antimicrobial resistance [25].

The European Food Safety Authority (EFSA) initiated a comprehensive, EU-wide monitoring program to continuously collect data on antimicrobial resistance (AMR) from livestock farms across the European Union, including poultry farms [9]. The assessment of resistance is based on clinical and epidemiological breakpoints established by the Clinical and Laboratory Standards Institute (CLSI) and the EUCAST. Given the widespread nature of AMR, reducing antibiotic usage and finding alternatives—such as plant essential oils [26], plant extracts [27], or antimicrobial peptides [28]—is of paramount importance. Propolis, with its complex flavonoid composition, has also been shown to effectively combat pathogens [29,30]. It has also been proven that medium-chain fatty acids and triglycerides adhere to the bacterial cell wall, causing damage and resulting in plasma leakage [31]. The poultry sector is among the largest consumers of antimicrobial agents and is a significant contributor to the spread of resistance [32]. To mitigate this, pharmacokinetic and pharmacodynamic studies of antibiotics are essential to determine appropriate therapeutic dosages and treatment durations [33]. Furthermore, the biosecurity status of the farms is also highly significant [3,34].

Clinical isolates provide a realistic representation of the AMR situation in pathogenic strains causing disease in humans. Therefore, we evaluated the antimicrobial susceptibility profile of *E. coli* strains isolated from clinical cases submitted to the Hungarian national reference laboratory over a one-year period, focusing on antibiotics of animal and public health significance.

## 2. Results

### 2.1. Regional Distribution and Origin of Samples Received

During the study, a total of 133 *E. coli* strains were examined. These strains were isolated from clinically symptomatic, deceased animals during necropsy at the national reference laboratory and provided to us as pure cultures. The majority of the strains originated from the Dél-Alföld region (54.9%), while 3.7% came from the Dél-Dunántúl region, 7.5% from the Észak-Alföld region, 11.3% from the Észak-Magyarország region, 15.0% from the Közép-Dunántúl region, 6.1% from the Közép-Magyarország region, and 1.5% from the Nyugat-Dunántúl region. The samples were collected from a total of 50 Hungarian municipalities and submitted to the national reference laboratory. The isolates were predominantly cultured from bone marrow (66.2%), while 27.1% were from the liver, 3.7% from the lungs, 1.5% from the oviduct, 0.8% from the joints, and 0.7% from the trachea.

### 2.2. Antimicrobial Susceptibility Testing

**Figure 1:**
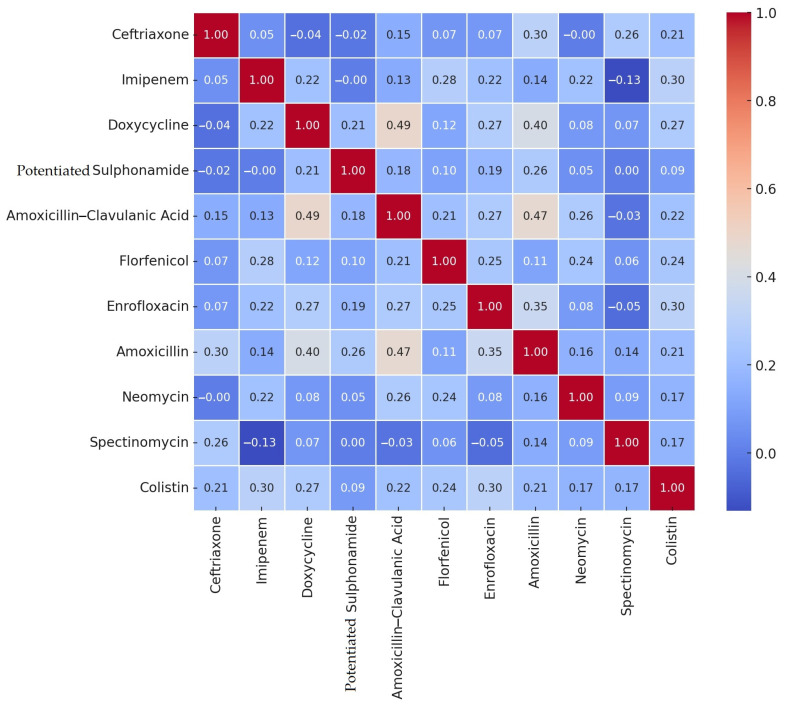
Plots of the correlation analysis based on the antimicrobial resistance profiles of the clinical *Escherichia coli* isolates (*n* = 133) received, plotted on a heat map by drug substance. The color key on the right side of the heatmap represents the correlation coefficient. The strongest positive correlation was observed between doxycycline and amoxicillin-clavulanic acid, as well as between amoxicillin and amoxicillin-clavulanic acid.

After determining the resistance rates for each antimicrobial agent based on clinical breakpoints, we performed a correlation analysis to explore the relationships between the levels of resistance of different agents (Figure 1). The strongest positive correlation was observed between doxycycline and amoxicillin-clavulanic acid (0.49; *p* < 0.001). A strong correlation was also found between amoxicillin and amoxicillin-clavulanic acid (0.47; *p* < 0.001). The strongest negative correlation, however, was observed between imipenem and spectinomycin (−0.13; *p* = 0.133).

**Figure 2:**
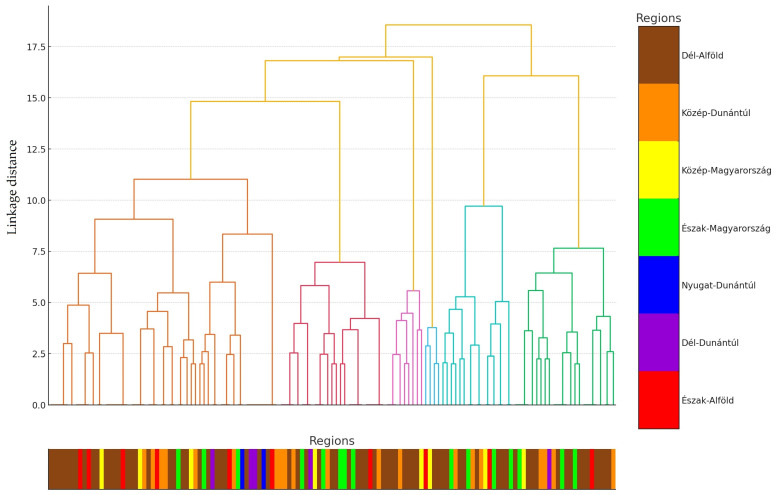
Individual-level cluster analysis of the clinical *Escherichia coli* isolates (*n* = 133) and a plot of the results on a dendrogram. For ease of reference, the regional origin of each sample was assigned a color, and a line was drawn below the horizontal axis in the color of the corresponding region. According to the cluster analysis, the similarity between samples is concentrated within certain regions; however, no sharp separation between clusters can be observed. This suggests that there is no clearly defined geographical pattern in antibiotic use or resistance profiles.

Subsequently, we performed a cluster analysis, visualizing the individual samples on a dendrogram (Figure 2). The primary objective of the dendrogram is to visually assess the relationships and clustering patterns among the samples based on antimicrobial resistance profiles. This clustering allows for the identification of potential groupings or regional effects within the data. Pairwise distances were calculated from the normalized data and subsequently assigned to the colors corresponding to the regions of origin of the samples. The regions represent the geographical origins of the isolates. This alignment likely reflects the similarity of samples within regions rather than indicating a perfect correlation.

**Figure 3:**
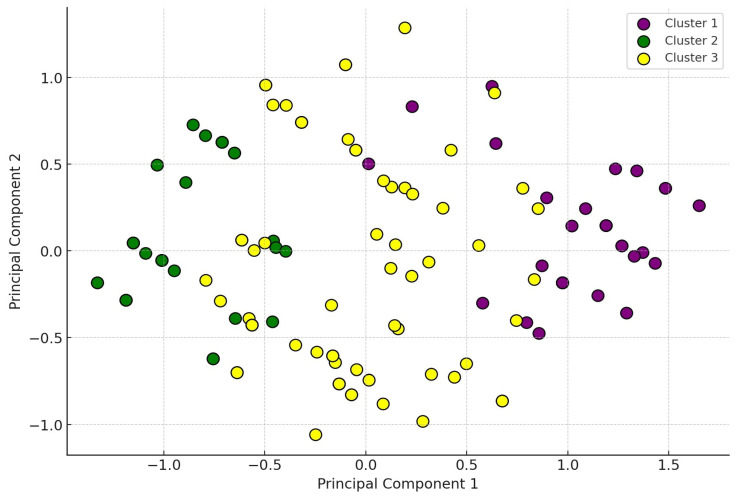
Distribution of data visualized in principal component analysis (PCA). The distribution of the data is uniform but well separated into three main clusters. There is no completely distinct separation between the clusters. Overlaps can be observed, particularly between Cluster 2 (green) and Cluster 3 (yellow). Cluster 1 (purple) is mostly located in the positive range, relatively isolated from the other clusters, while Cluster 2 (green) is primarily in the negative range, partially overlapping with Cluster 3. Cluster 3 (yellow) shows a broader distribution.

Subsequently, we conducted a principal component analysis (PCA), with the largest variance explained by variables representing the resistance profiles of the bacterial strains to the tested antibiotics (Figure 3). Each cluster represents a subset of samples that exhibit similar resistance profiles. This clustering allows for the identification of potential associations or distinct profiles within the dataset. The clusters reflect fundamental differences in antimicrobial resistance profiles. Strains classified within Cluster 1 exhibited high levels of resistance to doxycycline, potentiated sulfonamides, amoxicillin, and florfenicol. Strains belonging to Cluster 2 typically demonstrated substantial resistance to potentiated sulfonamides and neomycin, while showing moderate resistance to enrofloxacin and amoxicillin. In contrast, strains in Cluster 3 exhibited moderate resistance to potentiated sulfonamides and neomycin.

The MIC_50_ represents the minimum antimicrobial concentration required to inhibit the growth of 50% of microorganisms in a given population, while the MIC_90_ is the concentration needed to inhibit the growth of 90% of the population. These values are crucial for assessing the effectiveness of specific antibiotic or antimicrobial agents against various bacterial strains and help inform treatment strategies. Antibiotics with low MIC_50_ and MIC_90_ values for a specific pathogen are likely to be effective treatment options.

After determining the MIC values for each antimicrobial agent, we created a frequency distribution table (Table 1), which illustrates the data distribution. We also calculated the MIC_50_ and MIC_90_ values for each agent. Among the tested antimicrobial agents, only imipenem had both MIC_50_ and MIC_90_ values below the clinical breakpoint. Apart from imipenem, The MIC_50_ values were below the clinical breakpoint for amoxicillin-clavulanic acid, ceftriaxone, spectinomycin, doxycycline, enrofloxacin, and colistin.

We also compared our results with the ECOFF values defined by EUCAST. The ECOFF is a statistical metric used to distinguish wild-type (non-resistant) microorganisms from potentially resistant strains within a microbial population. It is calculated based on the MIC distribution of the antimicrobial agent and provides a critical reference point for assessing susceptibility and resistance trends. In our study, the MIC_50_ values for ceftriaxone and colistin were below their respective ECOFF thresholds.

**Figure 4:**
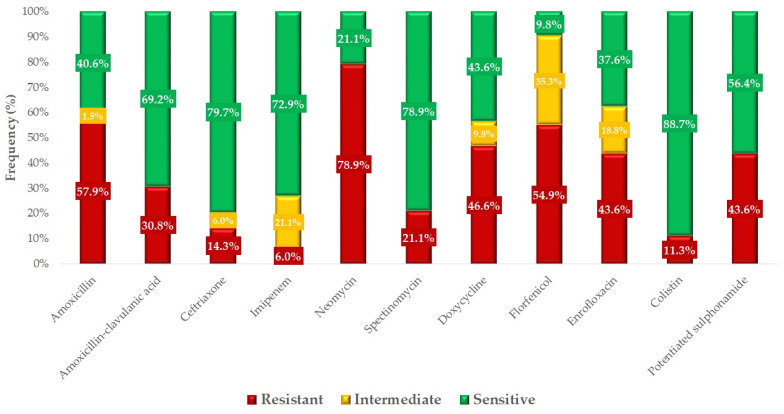
Antibiotic susceptibility profile of *Escherichia coli* strains (*n* = 133) isolated from clinical cases. The significantly different levels of resistance observed between amoxicillin and amoxicillin-clavulanic acid suggest that a substantial proportion of the strains produce beta-lactamase enzymes. Imipenem, ceftriaxone, and colistin demonstrated high efficacy; however, the high resistance to enrofloxacin (43.6%; 95% CI: 35.2–52.4%) is concerning and may be associated with the overuse of this compound in the poultry industry.

The frequency data for antimicrobial agents without clinical breakpoints are summarized in Supplementary Table S1. Based on the clinical breakpoints, the resistance levels to specific antimicrobial agents were determined (Figure 4). In total, 81.9% of the strains were found to be multidrug-resistant. The exact distribution of antimicrobial resistance among these strains is provided in the additional data file. The observed 57.9% resistance to amoxicillin (95% CI: 49.7–65.6%) and 30.8% resistance to amoxicillin-clavulanic acid (95% CI: 23.3–39.7%) suggest that some of the strains produce beta-lactamase, because the strains were significantly more sensitive to the combination antibiotic. The resistance rates to critically important agents such as ceftriaxone, enrofloxacin, and colistin are to a considerable extent. The highest resistance rate was observed against neomycin (78.9%; 95% CI: 71.4–85.1%).

**Figure 5:**
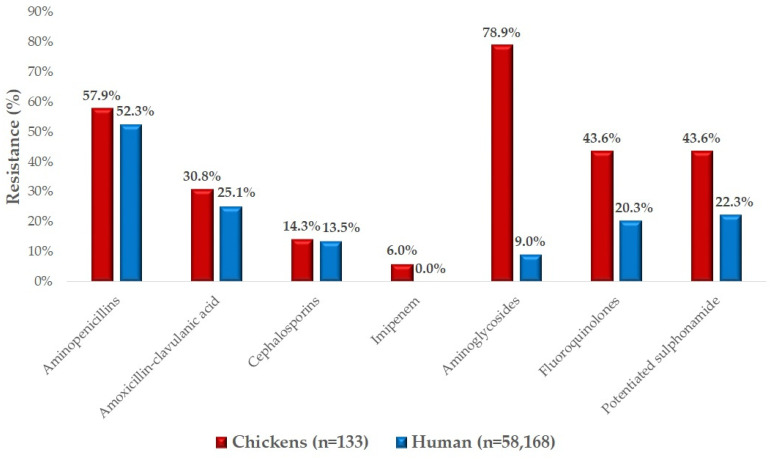
Reconciliation of clinical *Escherichia coli* strains isolated from domestic chickens and human susceptibility test results. A very similar level of resistance was observed for aminopenicillins, amoxicillin-clavulanic acid, and cephalosporins. However, in the poultry industry, as opposed to public health, the resistance rate against aminoglycosides was notably high.

We had the opportunity to compare our results with resistance data from human data (Figure 5). The comparison shows that the resistance levels for amoxicillin (57.9%; 95% CI: 49.7–65.6%) in veterinary medicine and ampicillin (52.3%; 95% CI: 51.6–52.4%) in human medicine were very similar. Although lower, the resistance rates to amoxicillin-clavulanic acid were also comparable (30.8%; 95% CI: 23.3–39.7% and 25.1%; 95% CI: 24.6–25.4%). Furthermore, resistance patterns for cephalosporin also exhibited similar trends across veterinary and human samples (14.3%; 95% CI: 9.0–21.3% and 13.5%; 95% CI: 13.7–14.3% respectively). However, for imipenem, while 6% (95% CI: 2.7–11.6%) resistance was observed in chicken isolates, no resistance was detected in human samples. In contrast, resistance rates to aminoglycosides, fluoroquinolones, and potentiated sulfonamides were consistently higher in veterinary medicine.

## 3. Discussion

This study tested the sensitivity of a total of 133 *E. coli* strains isolated from clinical cases in chickens, assessing their response to 15 antibiotics of veterinary and public health significance. Among these antibiotics, 11 had established clinical breakpoints, enabling us to calculate the percentage of resistance.

We observed a 57.9% resistance rate to amoxicillin (95% CI: 49.7–65.6%), which is higher than the 32% resistance to ampicillin reported by Hassan et al. [35]. Other studies reported varying resistance rates: Kaushik et al. found 89.4% resistance to penicillin and 80.4% to ampicillin [36], Much et al. recorded 19% resistance to ampicillin in organic systems and 33.8% in conventional systems [37], De Jong et al. reported resistance rates ranging from 32.7% to 65.3% for ampicillin [38], and Rivera-Gomis et al. documented a resistance rate of 30.8% [39]. Overall, our samples demonstrated higher resistance levels compared to the literature. Amoxicillin is widely used and one of the most frequently administered antibiotics. Its extensive use over decades, coupled with the high prevalence of beta-lactamase enzyme production in *E. coli*, likely contributed to the elevated resistance observed in our study.

In contrast to Majewski et al.’s findings of an 84.6% resistance rate to amoxicillin-clavulanic acid [40], we observed 30.8% (95% CI: 23.3–39.7%) resistance to amoxicillin-clavulanic acid, which indicates that a significant proportion of the strains we tested produce β-lactamase. Although the use of clavulanic acid is not approved in poultry, monitoring its in vitro efficacy remains important for both veterinary and public health.

Similarly, for ceftriaxone, we observed a resistance rate of 14.3% (95% CI: 9.0–21.3%), which is lower than the 28.2% reported by Kaushik et al. [36], or the 78.1% resistance to cefotaxime reported by Mandal et al. [41]. Ceftriaxone resistance can potentially be linked to the periodic use of ceftiofur in the poultry industry, as environmental resistance to cephalosporins can develop and spread rapidly. The continued and widespread increase in resistance to these antibiotics is concerning, but fortunately, this trend appears to be slowing due to the discontinuation of ceftiofur usage [42]. Nevertheless, commensal strains may serve as reservoirs for AMR, helping to maintain current resistance levels. This is significant because third- and fourth-generation cephalosporins are critically important antibiotics whose efficacy must be preserved.

The 6.0% (95% CI: 2.7–11.6%) resistance observed for imipenem also raises concerns, although Shaib et al. reported no resistance [43] and Moffo et al. described approximately 20% resistance [44]. The regional variations in resistance could stem not only from cross-resistance with other antibiotics but also from the frequently reported instability issues associated with imipenem [45]. These issues could be mitigated by investigating more stable antibiotics within the same class, such as meropenem or ertapenem.

For neomycin, we measured a resistance rate of 78.9% (95% CI: 71.4–85.1%), similar to the high levels of resistance reported by Majewski et al. (84.6%) [40]. However, Kaushik et al. and Rivera-Gomis et al. documented resistance rates for gentamicin of just 12.3% and 13%, respectively [36,39], while De Jong et al. found resistance rates ranging from as low as 0.9% to 7% [38]. Cross-resistance within the aminoglycosides is well-documented [46], highlighting the importance of preserving the sensitivity of gentamicin, a drug of significant public health importance. Although neomycin is among the most toxic aminoglycosides when administered parenterally, it can be safely used for oral administration. However, its selective pressure on the gut microbiome is considerable. For spectinomycin, 21.1% of the strains were resistant in our study, whereas Adelowo et al. reported a resistance rate of 47% [47].

Tetracyclines are among the oldest antibiotic classes and have been widely used in poultry. Their poor oral bioavailability has imposed significant environmental pressure, contributing to the widespread development of resistance. Accordingly, we observed 46.6% (95% CI: 38.1–55.3%) resistance to doxycycline which is similar to the 44% reported by Hassan et al. [35], the 62.1% resistance found by Rivera-Gomis et al. [39], and the resistance rates ranging from 41.3% to 67.5% reported De Jong et al. [38]. Mandal et al. reported an even higher resistance rate of 78.1% [41]. In contrast, Kaushik et al. documented just 17.4% resistance to tetracycline [36], Much et al. observed rates of 27.6% under organic farming conditions and 25.9% under conventional systems [37], and Majewski et al. found a resistance rate of 36% [40].

Our study also noted a high resistance rate to florfenicol (54.9%, 95% CI: 46.4–63.1%). This is concerning as other studies (Adelowo et al. [47]; Wang et al. [48]) found very low resistance rates. The high resistance in Hungary may be linked to the increased usage of florfenicol in veterinary settings. The importance of florfenicol has risen following the EU directive (2019/6 regulation) to reduce the use of critically important antimicrobials (3–4th generation cephalosporins, fluoroquinolones, and colistin).

For enrofloxacin, we measured a resistance rate of 43.6% 95% CI: 35.2–52.4%), which is higher than the 34.6% documented by Majewski et al. [40], the 32% reported by Hassan et al. [35], and the 6.5% resistance to ciprofloxacin observed by Kaushik et al. [36] but lower than the 69.1% resistance in conventional farming reported by Much et al. [37]. De Jong et al. reported ciprofloxacin resistance rates ranging from 7.3% to 21.3% [38]. Fluoroquinolones are critically important antimicrobials; unfortunately, they have been widely used in the poultry sector over the past decades, leading to widespread resistance. While ciprofloxacin is not used in poultry, a portion of enrofloxacin administered to poultry metabolizes into ciprofloxacin within the body, posing a potential risk to human health.

For colistin, we observed a resistance rate of 11.3% (95% CI: 6.6–17.9%), while Adelowo et al. reported no resistant strains [47], and Kempf et al. documented a resistance rate of 0.6% [49]. Colistin is also a critically important antimicrobial used for treating multidrug-resistant infections, and resistance to it is highly concerning. Efforts must focus on preserving its effectiveness, which requires limiting its use to rare, highly justified cases as a second-line treatment based on sensitivity testing results.

For potentiated sulfonamides, we found that 43.6% (95% CI: 35.2–52.4%) of the strains were resistant, a rate similar to that reported by Hassan et al. (38%) [35], De Jong et al. (27.5–49.7%) [38], and Majewski et al. (53.5%) [40]. This combination of active substances has been in use for a long time; however, its usage has declined in recent years. Over time, this reduction may allow the reemergence of wild-type strains, potentially leading to a decrease in resistance levels.

Although the sample size in this study did not allow us to evaluate the impact of location or type of production system, future studies with larger sample sizes should stratify samples accordingly. Previous studies have demonstrated significant differences in resistance profiles based on production types [50,51], which can be attributed to variations in rearing durations and differences in antibiotic usage.

We compared the resistance profiles of *E. coli* strains isolated from chickens with available data from human strains. The results for amoxicillin (57.9%; 95% CI: 49.7–65.6%) and ampicillin (52.3%; 95% CI: 51.6–52.4%) were very similar, as were those for amoxicillin-clavulanic acid. In human studies, Carmona-Cartaya et al. reported a 68% resistance rate to ampicillin and 28% for ampicillin-sulbactam [52], Carter et al. found 39.2% resistance to ampicillin and only 7.6% to amoxicillin-clavulanic acid [53], while Enyinnaya et al. documented 100% resistance to ampicillin and 67.8% to amoxicillin-clavulanic acid [54].

For enrofloxacin, we noted that resistance in chickens was 43.6% (95% CI: 35.2–52.4%), compared to 20.3% (95% CI: 19.7–20.3%) in human strains. However, Carmona-Cartaya et al. reported 55% resistance [52] and Enyinnaya et al. reported 54.08% resistance [54] to ciprofloxacin in human samples, whilst Carter et al. reported just 9% [53].

Regarding aminoglycosides, resistance in chickens was 78.9% (95% CI: 71.4–85.1%), significantly higher than the 9% (95% CI: 8.8–9.2%) resistance observed in human strains. Carter et al. found 7% resistance to gentamicin in humans [53], while Enyinnaya et al. reported 52% [54].

Resistance to potentiated sulfonamides was 43.6% (95% CI: 35.2–52.4%) in chickens, compared to 22.3% 95% CI: 21.7–22.3%) in human data. Interestingly, Carmona-Cartaya et al. reported only 1% resistance to potentiated sulfonamides [52].

For cephalosporins, resistance in chicken strains was 14.3% (95% CI: 9.0–21.3%), closely matching the 13.5% (95% CI: 13.7–14.3%) observed in human samples. In human studies, Chua et al. reported 5.7% resistance [55] and Carmona-Cartaya et al. reported 35% resistance to cefazolin [52], and Carter et al. reported 6% to cephalexin [53].

Horizontal gene transfer among commensal and pathogenic *E. coli* strains, facilitated by mobile genetic elements, may play a pivotal role in the dissemination of resistance genes across species and environments [19]. It is important to note that the sample size for human data was significantly larger, which may have influenced the results. Future studies in poultry should aim to include larger sample sizes to ensure more representative findings. Additionally, further investigation of multidrug-resistant strains using next-generation sequencing (NGS) is crucial for mapping the genetic basis of multidrug resistance. This could provide insights into the direction of gene flow, linking animal and human health and fostering the One Health approach.

## 4. Materials and Methods

### 4.1. The Origin of Samples and Human Data

The disease-deceased poultry were submitted to the National Food Chain Safety Office, Directorate of Veterinary Diagnostics, between February 2022 and May 2023 by veterinarians serving the farms. The samples were collected by veterinarians during routine diagnostic procedures as part of an agreement with the national reference laboratory, which provided the samples for further research purposes. The strains were isolated from deceased poultry samples submitted to the national reference laboratory, following standard necropsy procedures in accordance with professional guidelines [56]. We emphasize that the isolates were obtained from poultry carcasses that had undergone standard post-mortem examination by board-certified veterinary pathologists at the National Reference Laboratory. The majority of isolates were derived from bone marrow or liver samples, suggesting that a significant proportion of strains likely harbor virulence factors. The selection of the tested antibiotic agents was based on their significance in both veterinary and public health, with additional consideration to ensure the comprehensive coverage of as many antibiotic classes as possible.

From the samples collected during routine diagnostics, strains were isolated on ChromoBio^®^ Coliform (Biolab Zrt., Budapest, Hungary) medium, where *E. coli* formed blue-colored colonies with robust growth. The isolated blue colony-forming units were subsequently inoculated onto tryptone soy agar (Biolab Zrt., Budapest, Hungary) to establish colony cultures. The pure cultures were frozen at −80 °C using the Microbank™ system (Pro-Lab Diagnostics, Richmond Hill, ON, Canada).

Human resistance data were provided by the National Public Health and Pharmaceutical Center.

### 4.2. Minimum Inhibitory Concentration (MIC) Determination

The phenotypic expression of antimicrobial resistance (AMR) was assessed by determining the minimum inhibitory concentration (MIC) values for individual bacterial strains, following the methodology of the CLSI [57]. Breakpoints were also determined according to CLSI guidelines [57] and compared with the ECOFF defined by the EUCAST.

The bacterial strains stored at −80 °C were suspended in 3 mL of cation-adjusted Müller-Hinton broth (CAMHB; Biolab Zrt., Budapest, Hungary) the day before the assay and incubated at 37 °C for 18–24 h. The assays were conducted using 96-well microtiter plates (VWR International, LLC., Debrecen, Hungary). Except for the first column, all wells were filled with 90 µL of CAMHB. Stock solutions of the tested compounds (Merck KGaA, Darmstadt, Germany) at 1024 µg/mL were prepared according to CLSI guidelines [57]. From these, 180 µL of a 512 µg/mL solution diluted 1:1 with broth was added to the first column of the working plates, followed by a twofold serial dilution series. Excess 90 µL solution from the 10th column was discarded, leaving 90 µL in each well. Bacterial suspensions adjusted to a 0.5 McFarland standard using a nephelometer (ThermoFisher Scientific, Budapest, Hungary) were inoculated into the wells of the microtiter plates in reverse order from the 11th column onward, with 10 µL per well [57]. The evaluation was performed using the Sensititre™ SWIN™ automatic MIC reader (ThermoFisher Scientific, Budapest, Hungary) and VIZION system software v3.4 (ThermoFisher Scientific, Budapest, Hungary, 2024). The reference isolate used was *E. coli* (ATCC 25922).

### 4.3. Statistic Analysis

Statistical analysis was performed using R version 4.1.0 [58]. The normality of the data distribution was tested with the Shapiro–Wilk test. Data that did not follow a normal distribution were further analyzed using non-parametric tests. The resistance of each active substance was examined using the Kruskal–Wallis test [59], which does not assume a normal distribution and is suitable for comparing the medians of several sample groups—ideal for analyzing differences across various samples. A post hoc test was employed to determine specific correlations between groups. Pairwise comparisons were conducted using the Mann–Whitney U test [60] and *t*-tests with Bonferroni correction applied to adjust for inflated *p*-values resulting from multiple comparisons [61]. Correlation analysis is a statistical method aimed at determining the strength and direction of the relationship between two or more variables. Correlations indicate how variables change together but do not provide information about causality. A value of +1 signifies a perfect positive linear relationship, meaning that as one variable increases, the other increases proportionally. A value of 0 indicates no linear relationship between the variables, and a value of −1 represents a perfect negative linear relationship, where an increase in one variable corresponds to a proportional decrease in the other.

Cluster analysis is a statistical method used to group data such that elements with similar characteristics are placed in the same cluster, aiding in pattern recognition. During cluster analysis, data points are organized into clusters or groups, where elements within the same cluster are more similar to each other than to elements in different clusters. A dendrogram, a tree-structured diagram, visually represents the results of hierarchical clustering. The lower parts of the tree display individual data points, while the higher levels represent combinations of clusters.

PCA is a statistical method used for dimensionality reduction while preserving most of the information in the data. It is commonly used to reduce the number of descriptive variables in datasets, particularly large ones with significant redundancy among variables. The goal of PCA is to create fewer, independent (orthogonal) variables, called principal components, from the original data. These principal components are linear combinations that maximize the variance in the data. The components are ordered such that the first principal component explains the largest portion of the data’s variance, the second component is orthogonal to the first and explains the next largest portion of variance, and so on.

## 5. Conclusions

This study provides a comprehensive analysis of AMR in *E. coli* strains isolated from clinical cases in poultry in Hungary. Our findings reveal alarming levels of resistance to several critical antibiotics, particularly aminoglycosides and β-lactams, which are both used extensively in veterinary and human medicine. The observed resistance patterns highlight the potential risk of zoonotic transmission and emphasize the necessity for strict monitoring and prudent antibiotic usage practices.

The high resistance observed to neomycin (78.9%), as well as the significant levels of resistance to enrofloxacin and amoxicillin-clavulanic acid, reflect the long-term and widespread use of these drugs in poultry production. These results align with global trends but also reveal regional variations, which warrant further investigation.

A comparative analysis with human resistance data identified similar resistance patterns for several antibiotics, such as amoxicillin and cephalosporins. However, the notably higher resistance levels detected in isolates from chickens suggest that *E. coli* strains found in poultry may act as potential reservoirs for AMR, posing a significant public health risk.

To mitigate the AMR crisis, implementing antibiotic stewardship programs and well-organized biosecurity surveillance programs in poultry production, alongside exploring alternatives such as antimicrobial peptides or plant-based compounds, is critical. Although this study provides a detailed analysis of the phenotypic manifestation of antimicrobial resistance, it is important to note a limitation: the investigation did not extend to genetic analyses (neither PCR nor sequencing) to explore the underlying mechanisms of phenotypic resistance. Incorporating such analyses in future research could provide a comprehensive understanding of resistance genes and the mechanisms driving their expression.

Our study underscores the critical importance of the coordinated surveillance of AMR *E. coli* strains under the One Health framework, integrating animal and human health considerations. Future research should focus on identifying the drivers of resistance in poultry production and developing targeted interventions to mitigate the spread of AMR.

## Figures and Tables

**Table 1:** The frequency table of minimum inhibitory concentration (MIC) values for breakpoint-defined antimicrobial agents obtained from *Escherichia coli* samples originating from domestic chickens (*n* = 133). The upper row for each antimicrobial agent represents the count, while the lower row shows the corresponding percentage. Clinical breakpoints are indicated by vertical red lines.

Antibiotic	^1^ BP *	0.001	0.002	0.004	0.008	0.016	0.03	0.06	0.125	0.25	0.5	1	2	4	8	16	32	64	128	256	512	1024	MIC_50_	MIC_90_	^2^ ECOFF
	µg/mL																						µg/mL		
Enrofloxacin	^1^ 2	2	0	1	5	8	14	5	7	8	16	9	7	7	7	25	9	3					1	16	0.125
		1.5%	0.0%	0.8%	3.8%	6.0%	10.5%	3.8%	5.3%	6.0%	12.0%	6.8%	5.3%	5.3%	5.3%	18.8%	6.8%	2.3%							
Colistin	2	1	1	0	2	8	2	9	19	23	21	32	13	2									0.5	2	2
		0.8%	0.8%	0.0%	1.5%	6.0%	1.5%	6.8%	14.3%	17.3%	15.8%	24.1%	9.8%	1.5%											
Ceftriaxone	^1^ 4				1	8	29	34	12	8	8	6	8	3	1	5	0	2	0	1	2	5	0.06	16	0.125
					0.8%	6.0%	21.8%	25.6%	9.0%	6.0%	6.0%	4.5%	6.0%	2.3%	0.8%	3.8%	0.0%	1.5%	0.0%	0.8%	1.5%	3.8%			
Imipenem	^1^ 4						3	9	17	30	19	19	28	8									0.5	2	0.5
							2.3%	6.8%	12.8%	22.6%	14.3%	14.3%	21.1%	6.0%											
^3^ Potentiated sulphonamide	^1^ 4										2	4	13	20	16	15	5	3	30	0	7	18	16	1024	0.5
											1.5%	3.0%	9.8%	15.0%	12.0%	11.3%	3.8%	2.3%	22.6%	0.0%	5.3%	13.5%			
Doxycycline	^1^ 16										4	10	28	16	13	17	16	28	1				8	64	8
											3.0%	7.5%	21.1%	12.0%	9.8%	12.8%	12.0%	21.1%	0.8%						
Florfenicol	^1^ 16											1	0	12	47	31	12	19	4	7			16	64	16
												0.8%	0.0%	9.0%	35.3%	23.3%	9.0%	14.3%	3.0%	5.3%					
Amoxicillin	^1^ 32									1	2	0	14	19	18	2	42	1	5	8	12	9	32	512	8
										0.8%	1.5%	0.0%	10.5%	14.3%	13.5%	1.5%	31.6%	0.8%	3.8%	6.0%	9.0%	6.8%			
^4^ Amoxicillin-clavulanic acid	32										4	3	18	22	30	15	34	4	2	1			8	32	8
											3.0%	2.3%	13.5%	16.5%	22.6%	11.3%	25.6%	3.0%	1.5%	0.8%					
Neomycin	32											2	3	4	4	15	75	14	4	1	6	5	32	128	8
												1.5%	2.3%	3.0%	3.0%	11.3%	56.4%	10.5%	3.0%	0.8%	4.5%	3.8%			
Spectinomycin	128														2	9	72	22	10	5	6	7	32	256	64
															1.5%	6.8%	54.1%	16.5%	7.5%	3.8%	4.5%	5.3%			

* BP—breakpoint; ^1^ Clinical Laboratory Standard Institute (CLSI); ^2^ epidemiological cut-off value (EUCAST); ^3^ trimetophrime-sulphamethoxazole 1:19 ratio; ^4^ 2:1 ratio.

## Data Availability

The data presented in this study are available from the corresponding author upon reasonable request.

## Supplementary Materials

The following supporting information can be downloaded at: https://www.mdpi.com/article/10.3390/antibiotics14020176/s1, Table S1: Frequency table of the minimum inhibitory concentration (MIC) values (µg/mL) for agents without breakpoints in *Escherichia coli* samples derived from chickens (*n* = 133). The top row for each agent shows the count, while the bottom row shows the percentage. Additional excel file: Additional information on the strains included in the study.
