# Supplementary material for: Antimicrobial Susceptibility Profiles of Escherichia coli Isolates from Clinical Cases of Chickens in Hungary Between 2022 and 2023
Source: Antibiotics (Basel). 2025 Feb 11;14(2):176. doi: 10.3390/antibiotics14020176 (PMC11851984; doi:10.3390/antibiotics14020176)
Supplement: Supplementary file 1 [file antibiotics-14-00176-s001.zip › Supplementary materials.pdf]

Supplementary Table S1 Frequency table of the minimum inhibitory concentration (MIC) values (µg/mL) for agents without breakpoints in *Escherichia coli* samples derived from chickens (*n*=133). The top row for each agent shows the count, while the bottom row shows the percentage.

Antibiotic	0.001	0.002	0.004	0.008	0.016	0.03	0.06	0.125	0.25	0.5	1	2	4	8	16	32	64	128	256	512	1024	MIC_{50}	MIC_{90}
																						µg/mL	
**Tilozin**											1	0	0	2	0	0	66	1	9	37	17	64	1024
											0.8%	0.0%	0.0%	1.5%	0.0%	0.0%	49.6%	0.8%	6.8%	27.8%	12.8%		
**Tiamulin**																2	71	31	15	5	9	64	512
																1.5%	53.4%	23.3%	11.3%	3.8%	6.8%		
**Lincomycin**														1	0	0	66	0	0	18	48	64	1024
														0.8%	0.0%	0.0%	49.6%	0.0%	0.0%	13.5%	36.1%		
**Vancomycin**																67	1	8	36	16	5	32	512
																50.4%	0.8%	6.0%	27.1%	12.0%	3.8%		
